# Masquerading of COVID-19 Infection as Primary Mediastinal Large B-Cell Lymphoma

**DOI:** 10.4274/tjh.galenos.2020.2020.0340

**Published:** 2021-02-25

**Authors:** Ali Zahit Bolaman, Cem Selim, Can Karaman, İrfan Yavaşoğlu

**Affiliations:** 1 Aydın Adnan Menderes University Faculty of Medicine, Department of Hematology, Aydın, Turkey; 2 Aydın Adnan Menderes University Faculty of Medicine, Department of Radiology, Aydın, Turkey

**Keywords:** COVID-19 infection, Primary mediastinal large B-cell lymphoma

## To the Editor,

**Figure 1:**
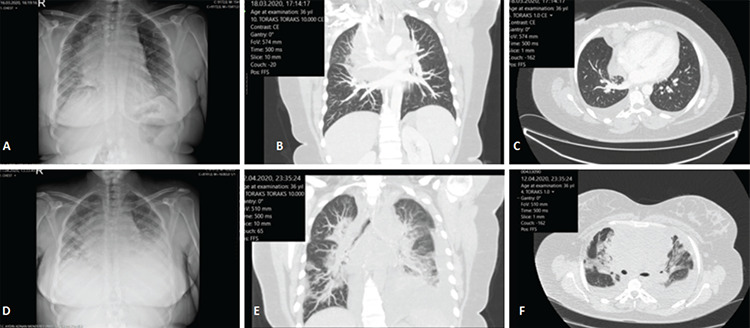
A computed tomography scan revealed a mediastinal mass surrounding the aorta and supraaortic vascular structures, compression of the superior vena cava, collapse and consolidation in right lung, and bilateral pleural effusion.

In March 2020, a female patient presented to the emergency department with cough and dyspnea. A computed tomography (CT) scan revealed a mediastinal mass surrounding the aorta and supraaortic vascular structures, compression of the superior vena cava, collapse and consolidation in the right lung, and bilateral pleural effusion (Figure 1). Lymph node biopsy suggested primary mediastinal large diffuse B-cell lymphoma (DLBCL). R-CHOP immunochemotherapy was started. Eighteen days after the beginning of chemotherapy, the patient was readmitted to the emergency department with the complaints of cough, dyspnea, and orthopnea for the past two days. The patient was restless and orthopneic, temperature was 37.3 °C, blood pressure was 137/102 mmHg, and pulse was 112 beats/min on physical examination. The patient’s respiratory rate was 32 breaths/min and oxygen saturation was 88% while breathing ambient air. The patient was referred to the hematology department by the emergency department’s staff because her symptoms were suggestive of mediastinal DLBCL. She had diffuse crackles in the middle zone of both lungs and breath sounds diminished bilaterally at the lower zone of both lungs. The patient’s hemoglobin level was 10.5 g/dL, white blood cell count 2880/µL, neutrophil count was 2150/µL, lymphocyte count was 320/µL, monocyte count was 400/µL, platelet count was 177000/µL, international normalized ratio was 0.9, fibrinogen was 583 mg/L (normal value: 180-350), D-dimer was 1307 ng/mL (normal value: <500), and lactate dehydrogenase was 804 U/L (normal value: <243). The CT scan was repeated. No changes were observed in the appearance of the mediastinal mass, but there was an increase in pleural effusion and ground-glass appearance with consolidation in various places in the lower zones of both lungs as assessed by CT scan (Figure 1, bottom row). Real-time polymerase chain reaction results (oropharyngeal swab from the patient) were positive for coronavirus disease (COVID-19). The patient was immediately transferred to the COVID-19 pandemic unit and hydroxychloroquine, azithromycin, and favipiravir treatment was immediately initiated [1,2]. Three days later, she was taken to intensive care and intubated because of acute respiratory distress syndrome. The patient died on the fourth day after intubation.

Symptoms such as cough, fever, and shortness of breath are common in primary mediastinal DLBCL. Therefore, this patient’s symptoms were linked with mediastinal DLBCL in the emergency room. It can be difficult to differentiate the symptoms of COVID-19 infection from those of mediastinal DLBCL. Therefore, health workers and especially emergency teams should be cautious not to confuse COVID-19 infection with DLBCL. Information on the comorbidity of non-Hodgkin’s lymphoma and COVID-19 is limited in the literature [3]. A case study was published of a patient with primary mediastinal large B-cell lymphoma and COVID-19 infection while our manuscript was being reviewed. The authors reported febrile neutropenia in a patient with primary mediastinal DLBCL after intensive immunochemotherapy (DA-EPOCH-R). They emphasized the importance of aggressive chemotherapy and rituximab for COVID-19 development in primary mediastinal DLBCL [4]. The American Society of Hematology did not suggest changing the treatment for patients who have already started treatment [5].
